# Acupuncture against the metabolic risk factors for stroke: A systematic review of systematic reviews

**DOI:** 10.1097/MD.0000000000030086

**Published:** 2022-09-02

**Authors:** Ying Xu, Da-yuan Zhong, Xiao-qian Liao, Xing-ping Wang, Jin-wen Ge, Wei-hui Xu

**Affiliations:** a Hunan University of Traditional Chinese Medicine, Changsha 410208, China; b The Third Hospital Affiliated to the Hunan University of Traditional Chinese Medicine, Zhuzhou, 412000, China; c Beijing University of Chinese Medicine, Beijing 102488, China.

**Keywords:** Acupuncture, AMSTAR-2, GRADE, review, stroke

## Abstract

This systematic review (SR) of SRs aims aimed to evaluate the current evidence of rehabilitation interventions in stroke patients after acupuncture treatment.

**Methods:**

Full-text SRs published in Chinese and English up to December 15, 2021 were searched in PubMed, Embase, Cochrane Library, CNKI, VIP, and Wanfang databases. The PRISMA statement and the assessment of multiple systematic reviews 2 (AMSTAR 2) scale were used to evaluate the quality of the included articles. The Grading of Recommendations, Assessment, Development and Evaluation (GRADE) system was employed to assess the outcome indicators for evidence quality evaluation.

**Results:**

A number of 42 publications were identified in this study. According to these articles, 4 metabolic areas were identified: systolic blood pressure, weight loss, glycemic index and cholesterol. The acupuncture is beneficial to improve the systolic blood pressure of patients, and the effect of acupuncture on diastolic blood pressure is better than that of sham acupuncture. The weight loss effect of acupuncture is better than that of lifestyle and western medicine. The improvement effect of acupuncture on body mass index (BMI) is also better than that of sham acupuncture. In the study of glycemic index of stroke patients, acupuncture significantly improved glycosylated hemoglobin and insulin sensitivity index compared with western medicine. In cholesterol-related research, acupuncture can effectively improve the content of triglycerides. However, studies on HDL and LDL show that acupuncture can significantly improve HDL, but has no significant effect on LDL.

**Conclusion:**

This review summarizes the available evidence and underpins findings of the acupuncture exhibited the therapeutic role in eliminating metabolic risk factors for stroke, including systolic blood pressure, weight loss, glycemic index and cholesterol. Acupuncture could have positive effects on a specific symptom, and the effects depend not only on intervention type but also on how and when the intervention is provided. And more prioritizing high-quality research in this field in the future is conducive to guiding clinical practice.

## 1. Introduction

Stroke is a global burden and affects not just individual but families, caregivers and society. Stroke is one of the major causes of the loss of life years in the world,^[1]^ and has become a major problem in the field of global public health. In 2016, 5.5 million^[2]^people worldwide died from stroke,and many initiatives are now reshaping stroke prevention, care, and rehabilitation in the country. According to the 2016 Global Burden of Disease Study, China had the highest estimated lifetime risk of stroke from age 25 years onwards of up to 39.3%, compared with 22.2% in Western Europe and 22.4% in high-income North America.^[3]^

Modern medicine for stroke prevention and control focuses on multiple risk factors. A recent study in the Lancet collated and analyzed risk factors for stroke and showed a high correlation with interventional risk factors such as hypertension, diabetes, and dyslipidemia.^[4]^ This is consistent with the consensus of experts on cardiovascular and cerebrovascular diseases in China.^[5]^ The consensus of China suggests that controlling the interventionable metabolic risk factors may be more effective in preventing the occurrence and development of stroke. The decline in mortality of cardiovascular diseases in western countries is closely related to the effective control of risk factors,^[6]^ which indicates a new feasible approach for the prevention of stroke.. More than thousands of years, acupuncture in China has been used as a traditional medical resource to ameliorate various diseases including stroke and other nervous system diseases.^[7]^ The effectiveness of acupuncture is closely related to the effective intervention of related risk factors of diseases. At present, there have been many systematic reviews to assess the intervention effect of acupuncture on related risk factors of diseases. Systematic evaluation is level evidence in the JBI(Joanna Briggs Institute) evidence classification. However, if the methodological quality of the system evaluation itself is not high, it will directly affect the credibility of its evidence.

Therefore, it is very important to carry out the reevaluation research on the system evaluation itself. This paper intends to reevaluate the current systematic evaluation of acupuncture intervention on metabolic risk factors of stroke, with a view to verifying the reliability and feasibility of acupuncture intervention on risk factors in the absence of high-quality evidence-based practice guidelines for stroke, and providing indirect reference evidence for acupuncture prevention and treatment of stroke.

## 2. Materials and Methods

### 2.1. Literature search and selection

Systematic search and screening procedures were carried out with the assistance of trained public health librarians. The following databases are systematically checked: the PubMed, Embase, Cochrane Library, CNKI, VIP, and Wanfang databases, which were set up until December 15, 2021. The references included in the systematic evaluation/meta-analysis were retrieved as a supplement. The Chinese search MeSH terms included acupuncture, body acupuncture, electroacupuncture, hypertension, regulating blood pressure, diabetes, blood sugar, insulin resistance, hyperlipidemia, lipid metabolism, obesity, overweight, systematic evaluation, meta-analysis, and meta-evaluation. The English search Mesh terms included acupuncture, electroacupuncture, hypertension, diabetes mellitus, meta-analysis, and systematic review, etc.

Inclusion criteria: (1) Types of article: all systematic evaluations or meta-analyses related to the metabolic risk factors for stroke treated with acupuncture. (2) Subjects: the diseases included hypertension, hyperlipidemia, obesity, and diabetes and were not limited to sex, age, race, nationality, or degree of disease.

(3) Intervention measures: the treatment group was treated with acupuncture (including acupuncture, electroacupuncture, or ear acupuncture) or with acupuncture that was mainly supplemented with other nonacupuncture therapy, and the control group was treated with placebo therapy (blank control or false acupuncture) or other nonacupuncture therapy.

Exclusion criteria: acupuncture as a nonmain intervention, such as acupoint compression, acupoint catgut embedding, moxibustion alone, and laser acupuncture; patients exhibiting metabolic high-risk factors complicated with other diseases; nonsystematic evaluation or meta-analysis; conference abstracts, letters, or reviews; failure to find full text or incomplete content; repeated literature; comparative studies between different acupuncture therapies; and plans for systematic evaluation.

### 2.2. Literature screening and extraction

Each of them carries on the the retrieval was performed by each researcher independently according to the preset retrieval strategy, and if the cross-check showed a difference, a third party analyzed the results. In cases with a lack of content and information, we attempted to contact the author. The extracted contents included the following: literature source, type of inclusion study, number of inclusion study, sample size, main outcome index, treatment group intervention, control group intervention, and bias risk assessment tool.

### 2.3. Quality evaluation

The PRISMA statement, AMSTAR 2^[8]^ and the GRADE tools^[9]^ were used for quality evaluation. The PRISMA consists of 7 parts: title, abstract, introduction, methods, results, discussion and funding, which contains 27 items with the judgment result of yes or no. The AMSTAR 2 evaluation tool contains 16 items, which involve the entire process of selecting topics, designing, registering, data extraction, statistical analysis, discussion of systematic evaluation, research questions, PICO elements of the inclusion criteria, systematic evaluation plan, type of research design included, literature retrieval strategy, literature screening, specific details of excluding the literature, bias risk assessment of the inclusion study, rationality of the statistical analysis, accuracy of the result interpretation, financial support, and conflict of interest. References were random or nonrandom.…, and entries 2, 4, 7, 9, 11, 13, and 15 were key entries that had a significant impact on the evaluation of the results. The GRADE evaluation tools mainly included 5 items: the limitation of the study, inconsistency of the results, indirect evidence, accuracy, and publication bias.

## 3. Results

### 3.1. Literature screening process and results

**Figure 1.**
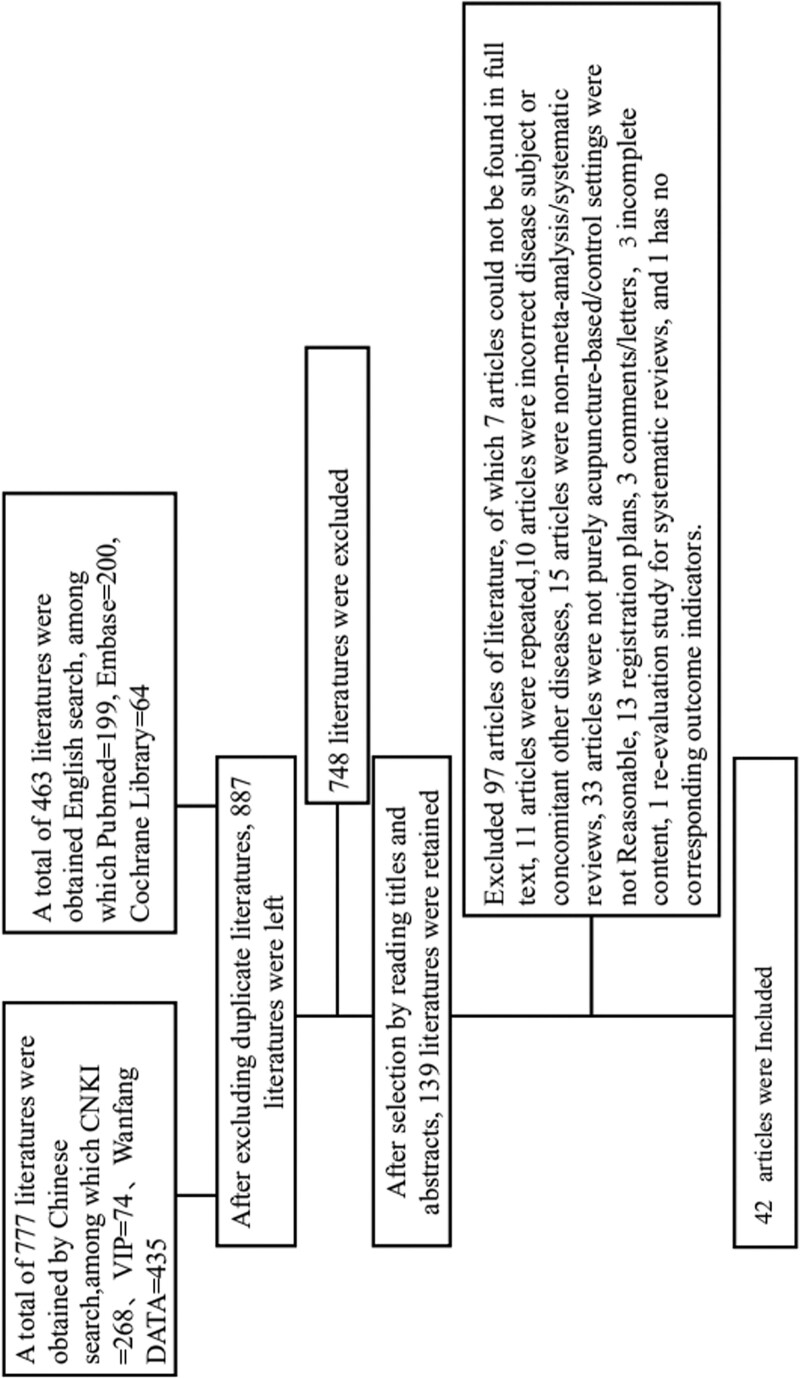
Identification: A total of 1240 documents were identified by systematically checked (777 literatures from Chinese databases, 463 literatures from English databases) each database Until December 15, 2021, search for relevant eligible randomized controlled trials with keywords or Mesh terms “acupuncture,” “body acupuncture,” “electroacupuncture,” “hypertension,” etc. In total, 887 articles left after removing duplicates. 2. Screening and extraction: By carefully reading the titles and abstracts of the literature, and according to the inclusion and exclusion criteria, the related literature was screened to determine whether it would be included in subsequent studies. If there is a dispute or a divergent issue, it will be resolved through internal consultation or discussion with a third party. A total of 139 articles were selected. 3. Included Full-text articles were assessed for eligibility, studies included in qualitative synthesis(systematic reviews) were 42.

A total of 1240 documents were identified through the systematic literature search, 887 articles left after removing duplicates. After consulting the title and abstract, 139 articles were selected. Our criteria included reexclusion of literature (n = 97) due to missing full text (n = 7), repeated literature (n = 11), incorrect disease subject or concomitant other diseases (n = 10), nonmeta-analysis/systematic evaluation (n = 15), nonpure needle-based/unreasonable control setting (n = 33), registration plan (n = 13),comments/letters (n = 3),and incomplete content(n = 3). The final number of publications included in this study was 42. The selection process is shown in Figure 1. A summary of the characteristics of the included articles is presented in Table 1.

**Table 1:** A summary of the characteristics of the literature included.

Literature	Type of study included	Number of studies included	Sample size	Male/female	Age	Treatment group intervention	Control group intervention	Outcomes
Hyangsook Lee 2009^[10]^	Randomized controlled trial	11	847	No limitation	40-72	Acupuncture and moxibustion + medicine	Fake acupuncture, fake acupuncture + medicine	Systolic and diastolic blood pressure
Chen C. 2019^[11]^	Randomized controlled trial	21	1943	Na	Mean age > 18	Acupuncture, auricular acupuncture, laser acupuncture, acupoint catgut embedding combined therapy	Western medicine	Blood sugar, 2 h blood sugar, glycosylated hemoglobin
Chen, 2018^[12]^	Randomized controlled trial	30	2107	1066/963/Not reported	Mean age > 18	Acupuncture and electroacupuncture	False acupuncture, western medicine, nontreatment, lifestyle intervention	Efficiency of blood pressure improvement, systolic and diastolic blood pressure
Cho, 2009^[13]^	Randomized controlled trial	31	3013	Na	no limitation	Acupuncture	Lifestyle interventions	Weight, obesity reduction efficiency
Dong-ZeLi 2014^[14]^	Randomized controlled trial	4	386	Na	Mean age > 18	Acupuncture and moxibustion + western medicine	Fake acupuncture, fake acupuncture + western medicine	Systolic and diastolic blood pressure
Junpeng Yao 2019^[15]^	Randomized controlled trial	12	1151	Na	>18/Not mentioned	Acupuncture, electroacupuncture + laser acupuncture, acupuncture + diet training, training,	Placebo, sham needle, diet training, training, no treatment	BMI, waistline reduction
KepeiZhang 2018^[10]^	Randomized controlled trial	21	1389	no limitation	no limitation	Acupuncture, electroacupuncture, body acupuncture, acupoint catgut embedding	False acupuncture, no treatment	BMI, weight, fat loss
Rong-Qiang Zhang 2017^[17]^	Randomized controlled trial	11	338 + 305	Na	>18	Acupuncture, electroacupuncture, body acupuncture	False acupuncture	BMI, fat reduction, waist reduction
Tan, 2019^[18]^	Randomized controlled trial	31	2649	no limitation	no limitation	Acupuncture	Life improvement, medication, sham acupuncture, and nontreatment	Systolic and diastolic blood pressure after treatment
Wang, 2013^[19]^	Randomized controlled trial	35	2539	no limitation	18-78	Acupuncture	Western medicine, sham acupuncture, lifestyle intervention	Systolic and diastolic blood pressure
Wu, 2019^[20]^	Randomized controlled trial	20	1639	no limitation	Mean age > 18	Acupuncture	Western medicine	Evaluation of dynamic equilibrium models for homa-IR and ISI.
Yang, 2018^[21]^	Randomized controlled trial	22	1744	no limitation	>18	Acupuncture	Western medicine	Systolic blood pressure; diastolic blood pressure
Zhao, 2015^[22]^	Randomized controlled trial	23	1788	no limitation	no limitation	Acupuncture, acupuncture + lifestyle intervention, acupuncture + western medicine	Western medicine, lifestyle	Efficiency of blood pressure improvement, systolic blood pressure, diastolic blood pressure, systolic blood pressure after treatment, diastolic blood pressure
Zhong, 2020^[23]^	Randomized controlled trial	8	403	no limitation	>18	Acupuncture, ear acupuncture, electroacupuncture, body acupuncture	False acupuncture, no treatment	BMI, weight, fat loss, waist reduction
Chang Xiaorong 2014^[24]^	Randomized controlled trial	9	733	Na	Na	Acupuncture	Western medicine	Total cholesterol, triglycerides, low-density cholesterol
Chen Hao 2019^[25]^	Randomized controlled trial and semirandomized controlled trial	64	5230	no limitation	Mean age > 18/Not mentioned	Acupuncture and moxibustion + western medicine	Western medicine	Systolic and diastolic blood pressure
Chen Xia 2016^[26]^	Randomized controlled trial (randomized controlled trial)	21	1929	no limitation	no limitation	Acupuncture	Western medicine	BMI, weight
Chen Yuyi 2017^[27]^	Randomized controlled trials and self-controlled trials	9	613	Na	Mean age > 18	Acupuncture + western Medicine	Western medicine	Efficiency of blood pressure improvement, systolic and diastolic blood pressure
Li Deping 2014^[28]^	Randomized controlled trial	7	754	Na	Na	Acupuncture	Western medicine	Obesity relief
Li Xiaohan 2015^[29]^	Randomized controlled trial s and Q- randomized controlled trial	5	317	no limitation	no limitation	Abdominal needle	Body needles/medicines	BMI, obesity relief efficiency
Lin Xiao Miao 2009^[30]^	Randomized controlled trial	8	1017	no limitation	no limitation	Acupuncture	Western medicine	BMI, weight
Liu Meilan 2015^[31]^]	Randomized controlled trial	9	733	no limitation	no limitation	Acupuncture and moxibustion, mild moxibustion, electroacupuncture, laser acupuncture	Western medicine	Total cholesterol, triglycerides, HDL, LDL
Liu Nan 2017^[32]^	Randomized and semi-randomized controlled trials	8	588	Na	Na	Acupuncture	Western medicine	Systolic pressure load, daytime mean systolic pressure, nocturnal mean systolic pressure, systolic pressure, diastolic pressure load, daytime mean diastolic pressure, nocturnal mean diastolic pressure, diastolic pressure
Ma Chunyan 2016^[33]^	Randomized controlled trial	15	1458	no limitation	>18	Acupuncture	Western medicine	Systolic pressure, systolic pressure, diastolic pressure, diastolic pressure
Ma Zhan 2012^[34]^	Randomized controlled trial and semi-randomized controlled trial	11	999	Na	Na	Acupuncture	Western medicine	Total cholesterol, triglycerides, HDL, LDL
Qian Yuxin 2013^[35]^	Randomized controlled trial	18	1473	no limitation	no limitation	Acupuncture, Acupuncture + routine Treatment	False acupuncture, routine treatment, positive drugs	Daytime systolic blood pressure, nocturnal systolic blood pressure, diurnal diastolic blood pressure, nocturnal diastolic blood pressure
Shi Liwei 2018^[36]^	Randomized controlled trial	11	970	411/519/Not reported	40-60	Acupuncture	Other therapies	BMI, fasting blood glucose, glycosylated hemoglobin, insulin sensitivity index
Tang Hongzhi 2011^[37]^	Randomized controlled trial	9	1087	no limitation	>18	Acupuncture	Western medicine	Blood pressure improves effective rate, systolic and diastolic blood pressure
Wang Feng 2019^[38]^	Randomized controlled trial	9	550	Na	Na	Acupuncture + western Medicine	Western medicine	Daytime systolic blood pressure, nocturnal systolic blood pressure, diurnal diastolic blood pressure, nocturnal diastolic blood pressure
Xia Yanyan 2015^[39]^	Randomized controlled trial	15	1082	145/937	18-60	Acupuncture	Lifestyle	BMI, weight
Xiao Gan-chen 2015^[40]^	Randomized controlled trial	15	1118	no limitation	no limitation	Acupuncture	Western medicine	Effective rate of hypotension, mean daytime systolic blood pressure, mean nocturnal systolic blood pressure, systolic blood pressure, diastolic blood pressure, mean daytime diastolic blood pressure, mean nocturnal diastolic blood pressure, diastolic blood pressure
Xing Chunguo 2015^[41]^	Randomized controlled trial	8	740	Na	Na	Acupuncture	Other therapies	Blood glucose and insulin sensitivity index
Yang Li Pan 2015^[42]^	Randomized controlled trial	16	1611	Na	Na	Abdominal needle	Western medicine	Obesity relief
Yu Hui 2013^[43]^	Randomized controlled and semi-randomized controlled trials, and nonrandomized clinical controlled trials,	18	1462	Na	Na	Acupuncture and moxibustion + western medicine	Fake Acupuncture and Western Medicine	Blood pressure improves total effective rate, systolic blood pressure after treatment, diastolic blood pressure after treatment
Yu Chi 2010^[44]^	Randomized, controlled, blind, clinical trial	8	958	Na	Na	Acupuncture	Western medicine	Obesity relief
Zhang Jiping 2017^[45]^	Randomized controlled trial	8	614	Na	Na	Acupuncture + western Medicine	Western medicine	Efficiency of blood pressure improvement, systolic and diastolic blood pressure
Zhang Lei 2017^[46]^	Randomized controlled trial	53	4459	no limitation	no limitation	Acupuncture and moxibustion + western medicine	Western medicine	Systolic and diastolic blood pressure
Zhang Lili 2013^[47]^	Clinical randomized controlled trial	11	1072	Na	Mean age:56	Acupuncture + western Medicine	Western medicine	Systolic and diastolic blood pressure after treatment
Zhang Yanjun 2014^[49]^	Randomized controlled and semi-randomized controlled clinical trials	13	1066	no limitation	33-78	Acupuncture and moxibustion + western medicine	Western medicine	Efficiency of blood pressure improvement, systolic and diastolic blood pressure
Zhao ran 2011^[50]^	Randomized controlled trial and semi-randomized controlled trial	18	1460	no limitation	no limitation	Acupuncture and moxibustion + Chinese medicine, acupuncture + western medicine, acupuncture and moxibustion, massage + electroacupuncture, acupuncture + behavioral therapy	Western medicine, behavioral therapy	Effective rate of blood pressure improvement, systolic blood pressure after treatment, diastolic blood pressure after treatment
Zhu Tao 2018^[48]^	Clinical randomized controlled trial	22	1758	Na	Na	Simple acupuncture/acupuncture + drugs/acupuncture + behavioral therapy	Drugs	No
Shan Zhongliang 2019^[51]^	Randomized controlled trial	5	380	Na	Na	Acupuncture	Lifestyle	Obesity relief

### 3.2. Quality assessment of included systematic reviews

Quality assessment of the 42 included articles showed the acceptable quality, with an obvious improvement of bias assessment methods and analyzing methods.The AMSTAR-2 evaluation tool was used to evaluate the quality of 42 included articles,^[10–51]^ and the results showed that 4 articles were evaluated as low quality and 38 articles were evaluated as very low quality, as shown in Table 2. The mean AMSTAR-2 score was 6.476 (Yes = 1, Partial Yes = 0, No = 0, complete score 16). The highest score is 12, and the lowest score is 1 (Table 1). Seven articles achieved good AMSTAR-2 score (“Y” ≥ 60%).^[11,13,21,22,33,37,40]^ Among the key items, the complete coincidence rate of item 2 was 0 (“Y” = 0%), because all the literature did not describe the research plan before implementation in detail, and only some literature indicated that the plan had been written in advance. This will be judged as Partial Yes. In item 4 and 7, literature^[11,13,19,21,22,33,37]^ had complete literature retrieval strategies, and supplementary retrieval was carried out for gray literature. Item 7, only one study^[21]^ provided a detailed list of exclusions and reasons. In item 9 and 26, articles,^[11,12,15,16,18–24,26,27,29,31–33,35–37,39,40,45–47,51]^ appropriate tools were used to assess the risk of bias in the included literature. In item 11and 18, articles^[11–13,16,20–23,27,30,32–34,39,40,44,45,50]^ were analyzed using appropriate statistical methods, and subgroup analysis was conducted for those with relatively high heterogeneity included. Items 13 and 24, artilcles^[10,11,13,14,16–23,29–31,33,34,36,37,39–41,44,49]^ fully discussed the risk of bias in the included articles and the possible causes. Items 15 and 20, articles^[11–13,15,17–19,25,26,28,33,34,36,37,40,43,44,46,48,51]^ were conducted for the research of migration (Table 2).

**Table 2:** Evaluation of methodological study quality.

Literature	Item 1	Item 2	Item 3	Item 4	Item 5	Item 6	Item 7	Item 8	Item 9	Item 10	Item 11	Item 12	Item 13	Item 14	Item 15	Item 16	Quality evaluation
Chen, C. 2019^[11]^	Y	PY	Y	Y	Y	Y	N	PY	Y	N	Y	Y	Y	Y	Y	Y	Low quality
Chen, H. 2018^[12]^	Y	PY	N	PY	Y	Y	PY	PY	Y	N	Y	Y	N	Y	Y	Y	Low quality
Cho, S.H. 2009^[13]^	Y	PY	N	Y	Y	Y	N	Y	PY	N	Y	Y	Y	Y	Y	Y	Low quality
Yang, J. 2018^[21]^	Y	PY	N	Y	Y	Y	Y	Y	Y	Y	Y	Y	Y	Y	N	N	Low quality
Hyangsook Lee 2009^[10]^	Y	PY	N	PY	Y	Y	PY	PY	PY	N	N	N	Y	N	N	Y	Very low quality
Dong-Ze Li 2014^[14]^	Y	N	N	PY	Y	Y	N	PY	PY	N	N	Y	Y	N	N	Y	Very low quality
Junpeng Yao 2019^[15]^	Y	PY	N	PY	Y	Y	N	Y	Y	N	N	N	N	N	Y	Y	Very low quality
Kepei Zhang 2018^[16]^	Y	N	N	PY	Y	Y	N	PY	Y	N	Y	N	Y	Y	N	Y	Very low quality
Rong-Qiang Zhang 2017^[17]^	Y	PY	Y	PY	Y	Y	N	Y	PY	N	N	Y	Y	N	Y	Y	Very low quality
Tan, X. 2019^[18]^	Y	PY	N	PY	Y	Y	N	PY	Y	N	N	N	Y	N	Y	Y	Very low quality
Wang, J. 2013^[19]^	Y	N	Y	Y	Y	Y	N	PY	Y	N	N	N	Y	Y	Y	Y	Very low quality
Wu, L. 2019^[20]^	Y	PY	N	PY	Y	Y	N	PY	Y	N	Y	Y	Y	Y	N	N	Very low quality
Zhao, X.F. 2015 [22]	Y	N	N	Y	Y	Y	N	Y	Y	N	Y	Y	Y	Y	N	Y	Very low quality
Zhong, Y.M. 2020^[23]^	Y	PY	N	PY	Y	Y	N	PY	Y	N	Y	N	Y	Y	N	Y	Very low quality
Chang Xiaorong 2014^[24]^	Y	N	N	PY	Y	Y	N	PY	Y	N	N	N	N	N	N	N	Very low quality
Chen Hao 2019^[25]^	Y	N	N	PY	Y	Y	N	PY	N	N	N	N	N	Y	Y	N	Very low quality
Chen Xia 2016^[26]^	Y	N	N	PY	Y	Y	N	PY	Y	N	N	N	N	N	Y	N	Very low quality
Chen Yuyi 2017^[27]^	Y	N	N	PY	N	N	N	Y	Y	N	N	N	N	N	N	N	Very low quality
Li Deping 2014^[28]^	Y	N	N	PY	Y	Y	N	N	N	N	N	N	N	N	Y	N	Very low quality
Li Xiaohan 2015^[29]^	Y	N	N	PY	Y	Y	N	PY	Y	N	N	N	Y	N	N	N	Very low quality
Lin Xiao Miao 2009^[30]^	Y	N	N	PY	Y	Y	N	PY	PY	N	Y	Y	Y	Y	N	N	Very low quality
Liu Meilan 2015^[31]^	Y	N	N	PY	Y	Y	N	PY	Y	N	N	N	Y	N	N	N	Very low quality
Liu Nan 2017^[32]^	Y	N	N	PY	Y	Y	N	PY	Y	N	Y	N	N	Y	N	N	Very low quality
Ma Chunyan 2016^[33]^	Y	N	N	Y	Y	Y	N	PY	Y	Y	Y	Y	Y	Y	Y	N	Very low quality
Ma Zhan 2012^[34]^	N	N	N	PY	N	N	N	PY	PY	N	Y	N	Y	Y	Y	N	Very low quality
Qian Yuxin 2013^[35]^	Y	N	N	PY	Y	Y	N	PY	Y	N	N	N	N	N	N	N	Very low quality
Shi Liwei 2018^[36]^	T	N	N	PY	Y	Y	PY	Y	Y	N	N	N	Y	N	Y	N	Very low quality
Tang Hongzhi 2011^[37]^	Y	N	N	Y	Y	Y	N	Y	Y	N	Y	Y	Y	Y	Y	N	Very low quality
Wang Feng 2019^[38]^	Y	N	N	PY	N	N	N	PY	PY	N	N	N	N	N	N	N	Very low quality
Summer heat 2015^[39]^	Y	N	N	PY	Y	Y	N	Y	Y	N	Y	Y	Y	Y	N	N	Very low quality
Xiao Gan-chen 2015^[40]^	Y	N	Y	PY	Y	Y	N	Y	Y	N	Y	Y	Y	Y	Y	N	Very low quality
Xing Chunguo 2015^[41]^	Y	N	N	PY	Y	Y	N	PY	PY	N	N	N	Y	N	N	N	Very low quality
Yang Li Pan 2015^[42]^	N	N	N	PY	Y	Y	N	PY	PY	N	N	N	N	N	N	N	Very low quality
Yu Hui 2013^[43]^	Y	N	N	PY	Y	Y	N	PY	PY	N	N	N	N	N	Y	N	Very low quality
Yu Chi 2010^[44]^	Y	N	N	PY	N	Y	N	PY	PY	N	Y	Y	Y	Y	Y	N	Very low quality
Zhang Jiping 2017^[45]^	Y	N	N	PY	Y	Y	N	PY	Y	N	Y	N	N	Y	N	N	Very low quality
Zhang Lei 2017^[46]^	Y	N	N	PY	Y	Y	N	PY	Y	N	N	N	N	N	Y	N	Very low quality
Zhang Lili 2013^[47]^	Y	N	N	PY	Y	Y	N	Y	Y	N	N	N	N	N	N	N	Very low quality
Zhu Tao 2018^[48]^	N	N	N	PY	N	N	N	PY	PY	N	N	N	N	N	Y	N	Very low quality
Zhang Yanjun 2014^[49]^	Y	N	N	PY	Y	Y	N	PY	PY	N	N	N	Y	N	N	N	Very low quality
Zhao ran 2011^[50]^	Y	N	N	PY	Y	Y	N	PY	PY	N	Y	N	N	N	N	N	Very low quality
Shan Zhongliang 2019^[51]^	Y	N	N	PY	Y	Y	N	PY	Y	N	N	N	N	N	Y	N	Very low quality

Note: Entry 1 refers to whether the question and criteria include PICO elements. Entry 2 is a systematic evaluation of whether the report was designed in advance and whether there are significant differences between the content of the report and the proposed program. Item 3 explains the choice of study design type. Entry 4 refers to whether a comprehensive literature retrieval strategy has been used in accordance with partial conformity. Entry 6 refers to the repeatability of the study screening and data extraction. Entry 7 is for the purpose of listing and proving that the exclusion partially complies. Item 8 describes in detail the contents of the NAI. Entry 9 is partially consistent with the use of appropriate methods to assess bias between natal studies. Item 10 provides information on the sources of funding for the NA study. Entry 11 is the suitability of the method for combining results. Entry 12 assesses the potential impact of the bias of the natal study on the meta-analysis results and other evidence synthesis, on interpreting and discussing the results of the systematic evaluation, and on understanding the bias of the NA study. Entry 14 is for a reasonable approach and to explain or discuss the heterogeneity observed in the evaluation results. Entry 15 is a quantitative merger and full investigation of publication bias to discuss its possible impact on the evaluation results. Entry 16 is for any potential conflict of interest reported and includes any funds received for systematic evaluation. Y: yes, N: no, PY: partially consistent.

### 3.3. Impact on blood pressure

A total of ten articles evaluated the effects of acupuncture on blood pressure improvement. Among them, six articles showed that acupuncture + western medicine exerted better antihypertensive effects than western medicine. Eight articles showed that acupuncture exerts a better antihypertensive effect than western medicine.Two articles showed that electroacupuncture exerts better antihypertensive effects than western medicine. Sixteen articles evaluated the reduction of systolic blood pressure due to acupuncture, western medicine, electroacupuncture, and acupuncture + lifestyle, and 3 articles showed that acupuncture + western medicine improved systolic blood pressure better than sham acupuncture + western medicine. Four articles showed that acupuncture was better than sham acupuncture in improving systolic blood pressure. Seven articles showed that acupuncture + western medicine improved systolic blood pressure better than western medicine. Seven articles showed that acupuncture improved systolic blood pressure better than western medicine.. Sixteen articles demonstrated that the effect of acupuncture on diastolic blood pressure was better than that of sham acupunctur. Acupuncture improved diastolic blood pressure better than western medicine. Acupuncture + western medicine improved diastolic blood pressure better than western medicine. Details are provided in Table 3.

**Table 3:** GRADE evaluation results of blood pressure-related indicators.

Indicators	Source of literature	Measures	MD	Quality of the evidence (GRADE)
Effective rate of blood pressure improvement	Zhang Lei 2017^[46]^	Acupuncture vs Western Medicine	1.10[1.03,1.17]	MODERATE ^1, 2^
Effective rate of blood pressure improvement	Chen, H. 2018^[12]^	Acupuncture vs Western Medicine	1.12 [0.98, 1.28]	LOW ^1,2,3^
Effective rate of blood pressure improvement	Tang Hongzhi 2011^[37]^	Acupuncture vs Western Medicine	1.57[0.95,2.61]	LOW ^1,3,4^
Effective rate of blood pressure improvement	Xiao Gan-chen 2015^[40]^	Acupuncture vs Western Medicine	1.2[1.06,1.35]	LOW ^1,3,4^
Effective rate of blood pressure improvement	Zhang Yanjun 2014^[49]^	Acupuncture vs Western Medicine	0.95[0.45,2.00]	LOW ^1,3,4^
Effective rate of blood pressure improvement	Zhao,X.F. 2015^[22]^	Acupuncture vs Western Medicine	1.14[0.70,1.85]	LOW ^1,2,4^
Effective rate of blood pressure improvement	Zhao ran 2011^[50]^	Acupuncture vs Western Medicine	1.44[0.76,2.75]	LOW ^1,2,4^
Effective rate of blood pressure improvement	Yu Hui 2013^[43]^	Acupuncture vs Western Medicine	1.04[0.95,1.14]	VERY LOW ^1,2,3,4^
Effective rate of blood pressure improvement	Zhao ran 2011^[50]^	Acupuncture + Traditional Chinese Medicine vs Western Medicine	1.78[0.62,5.17]	LOW ^1,2,4^
Effective rate of blood pressure improvement	Zhao,X.F. 2015^[22]^	Acupuncture + western medicine vs western medicine	4.19[1.65,10.67]	MODERATE ^1 4^
Effective rate of blood pressure improvement	Zhao ran 2011^[50]^	Acupuncture + western medicine vs western medicine	5.18[1.58,16.98]	MODERATE ^1 4^
Effective rate of blood pressure improvement	Zhang Lei 2017^[46]^	Acupuncture + western medicine vs western medicine	1.19[1.13,1.25]	MODERATE ^1 4^
Effective rate of blood pressure improvement	Chen, H. 2018^[12]^	Acupuncture + western medicine vs western medicine	1.17 [1.08, 1.27]	LOW ^1 4^
Effective rate of blood pressure improvement	Chen Yuyi 2017^[27]^	Acupuncture + western medicine vs western medicine	1.26[1.13,1.41]	LOW ^1,3,4^
Effective rate of blood pressure improvement	Zhang Jiping 2017^[45]^	Acupuncture + western medicine vs western medicine	4.07[2.45,6.76]	LOW ^1,3,4^
Effective rate of blood pressure improvement	Zhao ran 2011^[50]^	Acupuncture + Massage vs Western Medicine	5.21[1.28,21.24]	LOW ^1,2,4^
Effective rate of blood pressure improvement	Chen, H. 2018^[12]^	Life vs life + acupuncture	1.20 [1.05, 1.36]	LOW ^1 4^
Effective rate of blood pressure improvement	Zhao ran 2011^[50]^	Acupuncture + Behavioral Therapy vs Behavioral Therapy	2.30[1.02,5.19]	LOW ^1,2,4^
Effective rate of blood pressure improvement	Chen, H. 2018^[12]^	Electroacupuncture vs Western Medicine	0.94 [0.76, 1.16]	LOW ^1 4^
Effective rate of blood pressure improvement	Zhao ran 2011^[50]^	Electroacupuncture vs Western Medicine	0.86[0.29,2.55]	LOW ^1,2,4^
Shrink pressure	Liu Nan 2017^[32]^	Before acupuncture vs treatment	−15.41 [−22.65, −8.16]	MODERATE ^1 4^
Shrink pressure	Wang,J.2013^[19]^	Acupuncture vs Acupuncture + Western Medicine	−10.20 [−14.00, −6.40]	LOW ^1,2,4^
Shrink pressure	Ma Chunyan 2016^[33]^	Acupuncture vs Western Medicine	−7.24 [−11.07, −2.81]	LOW ^1,3,4^
Shrink pressure	Xiao Gan-chen 2015^[40]^	Acupuncture vs Western Medicine	−0.45 [−0.69, −0.21]	LOW ^1,3,4^
Shrink pressure	Chen, H. 2018^[12]^	Acupuncture vs Western Medicine	1.40 [−1.32,4.12]	LOW ^1,2,4^
Shrink pressure	Zhao,X.F. 2015^[22]^	Acupuncture vs Western Medicine	−0.56 [−3.02,1.89]	LOW ^1,2,4^
Shrink pressure	Zhang Lei 2017^[46]^	Acupuncture vs Western Medicine	−0.66 [−1.63, −0.29]	LOW ^1,2,4^
Shrink pressure	Wang,J.2013^[19]^	Acupuncture vs Western Medicine	−4.46 [−6.91, −2.02]	LOW ^1,2,4^
Shrink pressure	Tang Hongzhi 2011^[37]^	Acupuncture vs Western Medicine	0.84 [−3.69,5.36]	Extremely LOW ^1,2,3,4^
Shrink pressure	Wang,J.2013^[19]^	Acupuncture vs Lifestyle	−13.50 [−15.06, −11.94]	LOW ^1,2,4^
Shrink pressure	Wang,J.2013^[19]^	Acupuncture vs Fake Acupuncture + Western Medicine	−7.47 [−10.43, −4.51]	MODERATE ^1 4^
Shrink pressure	Dong-ZeLi 2014^[14]^	Acupuncture vs False Acupuncture	1.33 [−2.50,5.16]	Quality ^4^
Shrink pressure	Chen, H. 2018^[12]^	Acupuncture vs False Acupuncture	1.59 [−4.36,7.80]	MODERATE ^1 4^
Shrink pressure	HyangsookLee 2009^[10]^	Acupuncture vs False Acupuncture	−5 [−12, −1]	LOW ^1,2,4^
Shrink pressure	Wang,J.2013^[19]^	Acupuncture vs False Acupuncture	0.26 [−2.40,2.91]	LOW ^1,2,4^
Shrink pressure	Chen, H. 2018^[12]^	Acupuncture vs No Treatment	5.20 [−2.99,13.39]	LOW ^1,2,4^
Shrink pressure	Yang, J. 2018^[21]^	Acupuncture vs RAS inhibitors	−3.48 [−5.22, −1.74]	LOW ^1,2,4^
Shrink pressure	Chen, H. 2018^[12]^	Acupuncture + western medicine vs western medicine	9.80[2.95,16.65]	MODERATE ^1 4^
Shrink pressure	Zhang Jiping 2017^[45]^	Acupuncture + western medicine vs western medicine	−6.85 [−8.78, −4.39]	LOW ^1,3,4^
Shrink pressure	Zhang Yanjun 2014^[49]^	Acupuncture + western medicine vs western medicine	−9.5 [−13.65, −5.34]	LOW ^1,3,4^
Shrink pressure	Zhao,X.F. 2015^[22]^	Acupuncture + western medicine vs western medicine	−9.04 [−20.11,2.02]	LOW ^1,2,4^
Shrink pressure	Zhang Lei 2017^[46]^	Acupuncture + western medicine vs western medicine	−1.14 [−1.31, −0.96]	LOW ^1,2,4^
Shrink pressure	Zhu Tao 2018^[48]^	Acupuncture + western medicine vs western medicine	−3.14 [−4.61, −1.86]	VERY LOW ^1,2,3,4^
Shrink pressure	Chen Hao 2019^[25]^	Acupuncture + western medicine vs western medicine	NA	NA
Shrink pressure	Dong-ZeLi 2014^[14]^	Acupuncture + Western Medicine vs Fake Acupuncture + Western Medicine	−8.58 [−10.13, −7.03]	Quality ^4^
Shrink pressure	HyangsookLee 2009^[10]^	Acupuncture + Western Medicine vs Fake Acupuncture + Western Medicine	−8 [−10, −5]	MODERATE ^1 4^
Shrink pressure	Chen, H. 2018^[12]^	Acupuncture + Western Medicine vs Fake Acupuncture + Western Medicine	8.82[5.10,12.54]	MODERATE ^1 4^
Shrink pressure	Chen, H. 2018^[12]^	acupuncture + lifestyle vs lifestyle	10.38[6.72,14.04]	LOW ^1,2,4^
Shrink pressure	Zhao,X.F. 2015^[22]^	acupuncture + lifestyle vs lifestyle	−10.53 [−27.52,6.46]	LOW ^1,2,4^
Shrink pressure	Chen Yuyi 2017^[27]^	Acupuncture + Western Medicine vs Western Medicine	−9.98 [−15.87, −4.08]	VERY LOW ^1,2,3,4^
Shrink pressure	Chen, H. 2018^[12]^	Electroacupuncture vs Western Medicine	1.63 [−3.25,6.52]	MODERATE ^1 4^
Shrink pressure	Chen, H. 2018^[12]^	Electroacupuncture + Western Medicine vs Western Medicine	9.12[3.96,14.28]	LOW ^1,2,4^
Diastolic blood pressure	Liu Nan 2017^[32]^	Before acupuncture vs treatment	−11.46 [−18.72, −4.20]	LOW ^1,2,4^
Diastolic blood pressure	Zhao,X.F. 2015^[22]^	Acupuncture vs Western Medicine	−1.01 [−2.26,0.24]	MODERATE ^1 3^
Diastolic blood pressure	Yang, J. 2018^[21]^	Acupuncture vs Western Medicine	−1.64 [−2.81, −0.48]	MODERATE ^1 4^
Diastolic blood pressure	Ma Chunyan 2016^[33]^	Acupuncture vs Western Medicine	−2.81 [−4.55, −1.08]	LOW ^1,3,4^
Diastolic blood pressure	Tang Hongzhi 2011^[37]^	Acupuncture vs Western Medicine	0.88 [−115,7.09]	LOW ^1,3,4^
Diastolic blood pressure	Xiao Gan-chen 2015^[40]^	Acupuncture vs Western Medicine	−0.11 [−0.31,0.09]	LOW ^1,3,4^
Diastolic blood pressure	Wang,J.2013^[19]^	Acupuncture vs Western Medicine	−1.84 [−3.10, −0.58]	LOW ^1,2,4^
Diastolic blood pressure	Zhang Lei 2017^[46]^	Acupuncture vs Western Medicine	−0.61 [−1.02, −0.21]	LOW ^1,2,4^
Diastolic blood pressure	Wang,J.2013^[19]^	Acupuncture vs Lifestyle	−5.25 [−6.01, −4.49]	LOW ^1,2,4^
Diastolic blood pressure	Wang,J.2013^[19]^	Acupuncture vs Fake Acupuncture + Western Medicine	−4.22 [−6.26, −2.18]	MODERATE ^1 4^
Diastolic blood pressure	Dong-ZeLi 2014^[14]^	Acupuncture vs False Acupuncture	−0.18 [−3.98,3.62]	Quality ^4^
Diastolic blood pressure	Chen, H. 2018^[12]^	Acupuncture vs False Acupuncture	−0.01[−2.59, 2.57]	MODERATE ^1 4^
Diastolic blood pressure	HyangsookLee 2009^[10]^	Acupuncture vs False Acupuncture	−3 [−6,0]	LOW ^1,2,4^
Diastolic blood pressure	Wang,J.2013^[19]^	Acupuncture vs False Acupuncture	−1.04 [−2.56,0.47]	LOW ^1,2,4^
Diastolic blood pressure	Chen, H. 2018^[12]^	Acupuncture vs No Treatment	6.10 [1.27, 10.93]	LOW ^1,2,4^
Diastolic blood pressure	Chen, H. 2018^[12]^	Acupuncture + western medicine vs western medicine	3.31[4.67,10.96]	MODERATE ^1 4^
Diastolic blood pressure	Zhang Jiping 2017^[45]^	Acupuncture + western medicine vs western medicine	−4.44 [−6.19, −2.69]	LOW ^1,3,4^
Diastolic blood pressure	Zhang Yanjun 2014^[49]^	Acupuncture + western medicine vs western medicine	−0.16 [−2.52,2.19]	LOW ^1,3,4^
Diastolic blood pressure	Wang,J.2013^[19]^	Acupuncture + western medicine vs western medicine	−4.34 [−6.79, −1.90]	LOW ^1,2,4^
Diastolic blood pressure	Zhao,X.F. 2015^[22]^	Acupuncture + western medicine vs western medicine	−2.87 [−8.45,2.72]	LOW ^1,2,4^
Diastolic blood pressure	Zhang Lei 2017^[46]^	Acupuncture + western medicine vs western medicine	−1.10 [−1.63, −0.58]	LOW ^1,2,4^
Diastolic blood pressure	Zhu Tao 2018^[48]^	Acupuncture + western medicine vs western medicine	−4.50 [−6.45, −2.55]	VERY LOW ^1,2,3,4^
Diastolic blood pressure	Chen Yuyi 2017^[27]^	Acupuncture + western medicine vs western medicine	−6.06 [−9.61, −2.51]	VERY LOW ^1,2,3,4^
Diastolic blood pressure	Chen Hao 2019^[25]^	Acupuncture + western medicine vs western medicine	NA	NA
Diastolic blood pressure	HyangsookLee 2009^[10]^	Acupuncture + Western Medicine vs Fake Acupuncture + Western Medicine	−4 [−6, −2]	MODERATE ^1 4^
Diastolic blood pressure	Dong-ZeLi 2014^[14]^	Acupuncture + Western Medicine vs Fake Acupuncture + Western Medicine	−4.54 [−5.08, −4.00]	Quality ^4^
Diastolic blood pressure	Chen, H. 2018^[12]^	acupuncture + lifestyle vs lifestyle	5.74 [1.94, 9.54]	LOW ^1,2,4^
Diastolic blood pressure	Zhao,X.F. 2015^[22]^	acupuncture + lifestyle vs lifestyle	−7.52 [−15.06,0.02]	LOW ^1,2,4^
Diastolic blood pressure	Chen, H. 2018^[12]^	Electroacupuncture vs Western Medicine	−1.98[−4.58,0.62]	MODERATE ^1 4^
Diastolic blood pressure	Chen, H. 2018^[12]^	Electroacupuncture + Western Medicine vs Western Medicine	4.46[−0.25, 9.17]	LOW ^1,2,4^

1. indicates the limitations of the research, 2. indicates the inconsistency of the research results, 3. indicates indirect evidence, 4. indicates precision, 5. indicates publication bias.

### 3.4. Impact on obesity

The weight loss effect of electroacupuncture was better than that of lifestyle in 5 articles, and two items were statistically significant (one item of moderate quality and one item of low quality). The weight loss effect of acupuncture was better than that of western medicine (two items of low quality). The BMI improvement effect of electroacupuncture was better than that of sham acupuncture (two items of high quality). The BMI improvement effect of body acupuncture was better than that of sham acupuncture (one item of high quality). The BMI improvement effect of acupuncture was better than that of western medicine (one item of low quality and one item of very low quality). The detailed results are shown in Table 4.

**Table 4:** GRADE quality of obesity-related indicators.

Indicators	Source of literature	Measures	MD	Quality of the evidence(GRADE)
Weight	Chen Xia 2016^[26]^	Acupuncture vs Western Medicine	1.79[0.20,3.38]	LOW ^1,3,4^
Weight	Lin Xiao Miao 2009^[30]^	Acupuncture vs Western Medicine	1.94[1.73,2.16]	LOW ^1,3,4^
Weight	Cho,S.H. 2009^[13]^	Acupuncture vs Lifestyle	1.72[0.50,2.93]	MODERATE ^3 4^
Weight	Summer heat 2015^[39]^	Acupuncture vs Lifestyle	1.50,0.76,2.24]	LOW ^1,3,4^
Weight	Zhong,Y.M. 2020^[23]^	Acupuncture vs False Acupuncture	0.98[0.10,1.86]	MODERATE ^2 4^
Weight	Zhong,Y.M. 2020^[23]^	Acupuncture vs No Treatment	2.90[2.39,3.41]	MODERATE ^2 4^
Weight	Zhong,Y.M. 2020^[23]^	Body acupuncture vs sham acupuncture	3.00[1.51,4.49]	MODERATE ^2 4^
Weight	Zhong,Y.M. 2020^[23]^	Electroacupuncture vs Counterfeit Needle	3.78[2.66,4.90]	MODERATE ^2 4^
BMI	Lin Xiao Miao 2009^[30]^	Acupuncture vs Western Medicine	0.52[0.33,0.70]	LOW ^1,3,4^
BMI	Chen Xia 2016^[26]^	Acupuncture vs Western Medicine	0.15 [−1.00,1.31]	VERY LOW ^1,2,3,4^
BMI	Summer heat 2015^[39]^	Acupuncture vs Lifestyle	1.45[0.58,2.32]	LOW ^1,3,4^
BMI	Shi Liwei 2018^[36]^	Acupuncture vs other therapies	NA	NA
BMI	Rong-Qiang Zhang 2017^[17]^	Acupuncture vs False Acupuncture	0.48[0.40,0.57]	Quality ^4^
BMI	Zhong,Y.M. 2020^[23]^	Acupuncture vs No Treatment	1.52[0.42,2.61]	MODERATE ^2 4^
BMI	Rong-Qiang Zhang 2017^[17]^	Body acupuncture vs sham acupuncture	1.97 [−0.90,4.84]	MODERATE ^2 4^
BMI	Zhong, Y.M. 2020^[23]^	Body acupuncture vs sham acupuncture	1.97[1.19,2.75]	MODERATE ^2 4^
BMI	Li Xiaohan 2015^[29]^	Abdominal acupuncture vs other treatments	1.52[005,2.99]	VERY LOW ^1,2,3,4^
BMI	Zhong,Y.M. 2020^[23]^	Ear acupuncture vs false ear acupuncture	0.50[0.16,0.84]	MODERATE ^2 4^
BMI	Rong-Qiang Zhang 2017^[17]^	Electroacupuncture vs False Acupuncture	0.50[038,0.62]	Quality ^4^
BMI	Zhong,Y.M. 2020^[23]^	Electroacupuncture vs Counterfeit Needle	1.47[1.07,1.88]	Quality ^4^

1. indicates the limitations of the research, 2. indicates the inconsistency of the research results, 3. indicates indirect evidence, 4. indicates precision, 5. indicates publication bias.

### 3.5. Impact on blood glucose

Two articles investigated the blood glucose index and found that the hypoglycemic effect of acupuncture was significantly better than that of western medicine (one low quality). Ear acupuncture was significantly better than western medicine (one low quality). Two articles reported that acupuncture significantly improved glycosylated hemoglobin compared to western medicine (one low quality). Ear acupuncture also significantly improved glycosylated hemoglobin compared to western medicine (one low quality). Laser needle also seemed to be better than western medicine, but the difference was not statistically significant (one low quality). Three articles reported that acupuncture significantly improved the insulin sensitivity index compared to western medicine (one very low quality). The acupuncture-induced improvement in the insulin sensitivity index was significantly better than that of other therapies (one very low quality). The detailed results are shown in Table 5.

**Table 5:** Results of GRADE quality evaluation of blood glucose-related indicators.

Indicators	Source of literature	Measures	MD	Quality of the evidence (GRADE)
Blood glucose	Chen, C. 2019^[11]^	Acupuncture vs Western Medicine	−1.41 [−1.74, −1.07]	LOW ^1,2,4^
Blood glucose	Chen, C. 2019^[11]^	Auricular vs Western Medicine	−0.99 [−1.58, −0.39]	LOW ^1,2,4^
Blood glucose	Chen, C. 2019^[11]^	Finger Pressure vs Western Medicine	−0.19 [−0.72, 0.34]	LOW ^1,2,4^
Blood glucose	Chen, C. 2019^[11]^	Acupoint embedding vs Western medicine	−0.91 [−1.18, −0.64]	LOW ^1,2,4^
Blood glucose	Chen, C. 2019^[11]^	Combined treatment vs western medicine	−1.43 [−3.50, −0.87]	LOW ^1,2,4^
Blood glucose	Xing Chunguo 2015^[41]^	Acupuncture vs other therapies	−0.81 [−0.98, −0.64]	VERY LOW ^1,2,3,4^
Glycosylated hemoglobin	Chen, C. 2019^[11]^	Acupuncture vs Western Medicine	−1.21 [−1.78, −0.63]	LOW ^1,2,4^
Glycosylated hemoglobin	Chen, C. 2019^[11]^	Auricular vs Western Medicine	−0.37 [−0.64, −0.10]	LOW ^1,2,4^
Glycosylated hemoglobin	Chen, C. 2019^[11]^	Laser Acupuncture vs Western Medicine	−1.28 [−2.76, 0.20]	LOW ^1,2,4^
Glycosylated hemoglobin	Shi Liwei 2018^[36]^	Acupuncture vs other therapies	NA	NA
Insulin sensitivity index	Wu, L. 2019^[20]^	Acupuncture vs Western Medicine	0.36 [0.18, 0.53]	VERY LOW ^1,2,3,4^
Insulin sensitivity index	Xing Chunguo 2015^[41]^	Acupuncture vs other therapies	0.80 [0.36, 1.24]	VERY LOW ^1,2,3,4^
Insulin sensitivity index	Shi Liwei 2018^[36]^	Acupuncture vs other therapies	NA	NA

1. indicates the limitations of the research, 2. indicates the inconsistency of the research results, 3. indicates indirect evidence, 4. indicates precision, 5. indicates publication bias.

### 3.6. Impact on lipid

Three articles reported that acupuncture significantly improved total cholesterol better than western medicine (one moderate quality and two very low quality).Three articles reported that acupuncture improved triglyceride content significantly better than western medicine (two low quality and one very low quality). Two articles reported that acupuncture significantly improved HDL content better than western medicine (one moderate quality and one low quality). Three articles reported that acupuncture improved LDL results better than western medicine; one difference was statistically significant, and two differences were not statistically significant (two low quality and one very low quality). The specific results are shown in Table 6.

**Table 6:** GRADE quality evaluation results of total cholesterol.

Indicators	Source of literature	Measures	MD	Quality of the evidence(GRADE)
Total cholesterol	Liu Meilan 2015^[31]^	Acupuncture vs Western Medicine	−0.38 [−0.70, −0.06]	MODERATE ^1 4^
Total cholesterol	Chang Xiaorong 2014^[24]^	Acupuncture vs Western Medicine	−0.31 [−0.46, −0.15]	VERY LOW ^1,2,3,4^
Total cholesterol	Ma Zhan 2012^[34]^	Acupuncture vs Western Medicine	−0.07 [−0.10, −0.04]	VERY LOW ^1,2,3,4^
Total cholesterol	Liu Meilan 2015^[31]^	VSvs Western Medicine by Laser Acupoint Acupuncture	0.79 [0.27, 1.30]	LOW ^1,2,4^
Total cholesterol	Liu Meilan 2015^[31]^	Electroacupuncture vs Western Medicine	0.93 [0.66, 1.19]	LOW ^1,2,4^
Triglyceride	Chang Xiaorong 2014^[24]^	Acupuncture vs Western Medicine	0.13 [0.10, 0.16]	LOW ^1,3,4^
Triglyceride	Ma Zhan 2012^[34]^	Acupuncture vs Western Medicine	0.05 [0.02, 0.08]	VERY LOW ^1,2,3,4^
Triglyceride	Liu Meilan 2015^[31]^	Acupuncture vs Western Medicine	0.46 [0.13, 0.78]	LOW ^1,2,4^
Triglyceride	Liu Meilan 2015^[31]^	VSvs Western Medicine by Laser Acupoint Acupuncture	−5.60 [−6.73, −4.46]	LOW ^1,2,4^
Triglyceride	Liu Meilan 2015^[31]^	Electroacupuncture vs Western Medicine	−5.32 [−5.85, −4.78]	LOW ^1,2,4^
High density lipoprotein	Ma Zhan 2012^[34]^	Acupuncture vs Western Medicine	−0.16 [−0.17, −0.15]	VERY LOW ^1,2,3,4^
High density lipoprotein	Liu Meilan 2015^[31]^	Acupuncture vs Western Medicine	3.51 [1.48, 8.32]	MODERATE ^1 4^
High density lipoprotein	Liu Meilan 2015^[31]^	VSvs Western Medicine by Laser Acupoint Acupuncture	2.42 [0.55, 10.70]	LOW ^1,2,4^
High density lipoprotein	Liu Meilan 2015^[31]^	Electroacupuncture vs Western Medicine	1.08 [0.68, 1.69]	LOW ^1,2,4^
Low-density lipoprotein	Chang Xiaorong 2014^[24]^	Acupuncture vs Western Medicine	−0.02 [−0.15, 0.12]	LOW ^1,3,4^
Low-density lipoprotein	Ma Zhan 2012^[34]^	Acupuncture vs Western Medicine	0.34 [0.31, 0.37]	VERY LOW ^1,2,3,4^
Low-density lipoprotein	Liu Meilan 2015^[31]^	Acupuncture vs Western Medicine	−0.13 [−0.40, 0.14]	LOW ^1,2,4^
Low-density lipoprotein	Liu Meilan 2015^[31]^	VSvs Western Medicine by Laser Acupoint Acupuncture	−0.79 [−1.31, −0.27]	LOW ^1,2,4^
Low-density lipoprotein	Liu Meilan 2015^[31]^	Electroacupuncture vs Western Medicine	−0.32 [−0.57, −0.07]	LOW ^1,2,4^

1. indicates the limitations of the research, 2. indicates the inconsistency of the research results, 3. indicates indirect evidence, 4. indicates precision, 5. indicates publication bias.

## 4. Discussion

The systematic review synthesizes the existing literature on acupuncture treatment of stroke and comprehensively summarizes the effects of various acupuncture combined with other interventions, which can be used as the basis for personalized treatment of stroke in clinical practice.

Stroke is an acute cerebrovascular disease, which occurs more often in men over the age of 40. Stroke can cause ischemic or hemorrhagic changes in the brain, and cause severe cognitive impairment and limb dysfunction.^[52]^ At present, the incidence of complications caused by drug and surgical treatment for stroke is high, and the improvement of patients’ prognosis is limited.^[53–56]^ Kim Ka found that cerebrovascular injury and the structure and function of the blood-brain barrier were changed under the action of various risk factors.^[57]^ The oxidative stress of tissues caused by acute ischemia and hypoxia and the local inflammation caused by harmful factors entering the brain parenchyma through the blood-brain barrier cause damage to the nervous system. Cerebrovascular disease, which is mainly caused by chronic vascular endothelial injury caused by cerebrovascular metabolic risk factors, can directly cause cerebrovascular injury and secondary nerve injury.^[58]^ Therefore, intervention of metabolic risk factors such as hypertension, hyperglycemia, dyslipidemia and obesity is an important means to prevent stroke, and this view has also been affirmed by expert consensus.^[5]^

The main outcome indicators of acupuncture intervention all reflect the good efficacy of acupuncture on metabolic risk factors. Results form this study shows that the therapeutic effect of acupuncture intervention on the basis of the original treatment is more objective to a certain extent on the influencing factors of stroke. Related studies have shown that acupuncture is beneficial to improve the systolic blood pressure of patients, and the effect of acupuncture on diastolic blood pressure is better than that of sham acupuncture. In some studies, it has also been found that obesity is an influencing factor. The weight loss effect of acupuncture is better than that of lifestyle and western medicine. The improvement effect of acupuncture on BMI is also better than that of sham acupuncture. In the study of glycemic index of stroke patients, acupuncture significantly improved glycosylated hemoglobin and insulin sensitivity index compared with western medicine. However, there is no evidence that the effect of acupuncture with laser needle is better than that of western medicine. In cholesterol-related research, acupuncture can effectively improve the content of triglycerides. Besides, studies on HDL and LDL show that acupuncture can significantly improve HDL, but has no significant effect on LDL. These results can be used as a foundation for individualized treatment and aid health care professionals in meeting patients’individual needs and preferences.

However, the GRADE evaluation demonstrated that the results regarding blood pressure, obesity, blood sugar, and blood lipids are mainly of low quality and that the evaluation of blood sugar and blood lipids is not of high quality. The reason is that the limitations and accuracy of research are mainly related to blood pressure and that these studies have inconsistent research results and insufficient indirect evidence. The research results on obesity are inconsistent and inaccurate, and the research regarding blood glucose and blood lipid items is limited, with inaccurate and inconsistent results. This suggests that the sample size included in the systematic evaluation of metabolic risk factors of acupuncture intervention was small. The included systematic reviews of blood glucose and lipids were also downgraded due to limitations, indicating that the relevant clinical trials were not rigorously designed in terms of blindness, allocation concealment, and randomization, which reduced the credibility of the level of evidence.

## 5. Strengths and limitations

It is greatly needed in the area of stroke treatment and rehabilitation, which has a large number of inconsistent studies with acupuncture interventions and results. One way to make evidence available to clinical decision makers is by providing them with a summary of available evidence through a SR of SRs. Through such an approach, our this study provides a comprehensive evidence base by the standardized retrieval process, and clear and detailed inclusion and exclusion criteria during the study. Besides, there are some limitations in this study. Many of the included literature were of low quality, which may affect the accuracy of the research results. The outcome indicators of the included publications are not uniform, and thus, this study cannot be quantitatively and accurately compared, and there are errors. A potential limitation of this SR of SRs is that when including both SR and meta-analyses there is a risk that the same studies may have been included in more than one SR. Therefore, we chose not to draw conclusions on the number of SRs presented within each area or based on the study design. However, the inclusion of both SRs and meta-syntheses enables a broader scope and a more comprehensive approach to acupuncture interventions of stroke treatment and rehabilitation compared with other SRs. Thus, although there are some limitations in this paper, this paper has systematically and completely collected relevant literature by combining multiple databases, so this paper still has a certain guiding significance.

## 6. Conclusions

This review summarizes the available evidence and underpins findings of the acupuncture exhibited the therapeutic role in eliminating metabolic risk factors for stroke, including systolic blood pressure, weight loss, glycemic index and cholesterol. Acupuncture could have positive effects on a specific symptom, and the effects depend not only on intervention type but also on how and when the intervention is provided. And more prioritizing high-quality research in this field in the future is conducive to guiding clinical practice.

## Author contributions

Study concept and design: Xu.Y. Ge.J-W.and Xu.W-H. All authors contributed significantly to the acquisition, analysis, and interpretation of data; and critical revision of the manuscript for important intellectual content. All authors reviewed the results and approved the final version of the manuscript.

## References

[1] Christopher J Murray L. Global burden of 369 diseases and injuries in 204 countries and territories, 1990-2019: a systematic analysis for the global burden of disease study 2019. Lancet. 2014;396:1204–22. 10.1016/S0140-6736(20)30925-9 PMC7567026 33069326

[2] GBD 2016 Stroke Collaborators. Global, regional, and national burden of stroke, 1990-2016: a systematic analysis for the global burden of disease study 2016. Lancet. 2019;18:439–58. 10.1016/S1474-4422(19)30034-1 PMC6494974 30871944

[3] Feigin VL Nguyen G Cercy K; GBD 2016 Lifetime Risk of Stroke Collaborators. Global, regional, and Country-Specific lifetime risks of stroke, 1990 and 2016. N Engl J Med. 2018;379:2429–37. 30575491 10.1056/NEJMoa1804492 PMC6247346

[4] Bruce CVC Khatri P. Stroke. Lancet. 2020;396:129–42. 32653056 10.1016/S0140-6736(20)31179-X

[5] Wang XP Gao CY Li MW. Expert consensus on assessment, detection and intervention of common risk factors for cardiovascular and cerebrovascular diseases. ChinJ Appl Diagnosis Treat. 2021;35:541–51.

[6] Ford ES Ajani UA Croft JB. Explaining the decrease in us deaths from coronary disease, 1980-2000. Surv Anesthesiol. 2007;51:326. 10.1056/NEJMsa053935 17554120

[7] Xia Y Ding GH Wu GC. Current research in acupuncture. Springer. 2013:559–99.

[8] Tao H Yang LT Ping A. Quality evaluation tool AMSTAR 2 for systematic reviews of random or non-random control research Interpretation. Chin J Evid Based Med. 2018;18:101–8.

[9] Chen YL Yao L Susan N. The necessity and precautions of GRADE in systematic review. Chin J Evid Based Med. 2013;13:1401–4.

[10] Lee H Kim SY Park J. Acupuncture for lowering blood pressure: systematic review and meta-analysis.. Am J Hypertens. 2009;22:122–8. 19008863 10.1038/ajh.2008.311

[11] Chen C Liu J Sun MX. Acupuncture for type 2 diabetes mellitus: a systematic review and meta-analysis of randomized controlled trials. Complement Ther Clin Pract. 2019;36:100–12. 31383426 10.1016/j.ctcp.2019.04.004

[12] Chen H Shen FE Tan XD. Efficacy and safety of acupuncture for essential hypertension: a meta-analysis. Med scie monit. 2018;24:2946–69. 10.12659/MSM.909995 PMC5963739 29735972

[13] Cho SH Lee JS Thabane L. Acupuncture for obesity: a systematic review and meta-analysis. Int J Obes. 2019;33:183–96. 10.1038/ijo.2008.269 19139756

[14] Li DZ Zhou Y Yang YN. Acupuncture for essential hypertension: a meta-analysis of randomized sham-controlled clinical trials. Evid Based Complement Alternat Med. 2014;2014:279478. 24723957 10.1155/2014/279478 PMC3960742

[15] Yao JP He ZQ Chen Y. Acupuncture and weight loss in Asians: a PRISMA-compliant systematic review and meta-analysis. Medicine. 2019;98:e16815. 31415397 10.1097/MD.0000000000016815 PMC6831107

[16] Zhang KP Zhou SG Wang CY. Acupuncture on obesity: clinical evidence and possible neuroendocrine mechanisms. Evid Based Complement Alternat Med. 2018;2018:6409389. 30013603 10.1155/2018/6409389 PMC6022277

[17] Zhang RQ Tan J Li FY. Acupuncture for the treatment of obesity in adults: a systematic review and meta-analysis.. Postgrad Med J. 2017;93: 743–751. 28689171 10.1136/postgradmedj-2017-134969

[18] Tan X Pan Y Su W. Acupuncture therapy for essential hypertension: a network meta-analysis. Ann Transl Med. 2019;7:266. 31355233 10.21037/atm.2019.05.59 PMC6614319

[19] Wang J Xiong X Liu W. Acupuncture for essential hypertension. Int J Cardiol. 2013;169:317–26. 24060112 10.1016/j.ijcard.2013.09.001

[20] Wu L Chen X Liu Y. Role of acupuncture in the treatment of insulin resistance: a systematic review and meta-analysis. Complement Ther Clin Pract. 2019;37:11–22. 31445362 10.1016/j.ctcp.2019.08.002

[21] Phillips J. Acupuncture for hypertension. Res Nurs Health. 2020;43:294–5. 31898809 10.1002/nur.22004

[22] Zhao XF Hu HT Li JS. Is acupuncture effective for hypertension? a systematic review and meta-analysis. PLoS One. 2015;10:e0127019. 26207806 10.1371/journal.pone.0127019 PMC4514875

[23] Zhong YM Luo XC Chen Y. Acupuncture versus sham acupuncture for simple obesity: a systematic review and meta-analysis. Postgrad Med J. 2020;96:221–7. 32015189 10.1136/postgradmedj-2019-137221 PMC7146934

[24] Chang XR. Evidence-based study on acupuncture and moxibustion for hyperlipidemia. Chin Assoc Acupunct Moxibustion. 2014:8–13.

[25] Chen H. Construction of evidence system of acupuncture and moxibustion for essential hypertension based on reticular analysis. Nanjing Univ Chin Med. 2019.

[26] Chen X Huang W Deng J. A meta analysis of the effect of acupuncture at renying point on essential hypertension. J Clin Acupunct Moxibustion. 2016;32:64–9.

[27] Liu N Fan XN Meng ZH. A meta analysis of the effect of acupuncture at renying point on essential hypertensio. Henan Tradit Chin Med. 2017;37:1282–7.

[28] Li DP Kong LH Qin R. Meta analysis of clinical effect of acupuncture and moxibustion on simple obesity. J Hubei Univ Tradit Chin Med. 2014;16:100–2.

[29] Li XH. Systematic evaluation of li xiaohan’s abdominal acupuncture therapy for simple obesity. Chengdu Univ TCM. 2015.

[30] Lin XM Li B Du YH. Systematic evaluation of clinical effect of acupuncture on simple obesity. Chin Acupuncture Moxibustion. 2009;29:856–60. 19873927

[31] Liu ML Zhang GS Li CW. Systematic evaluation of randomized controlled clinical trial of acupuncture and moxibustion for hyperlipidemia. Liaoning J Tradit Chin Med. 2015;42:2065–70.

[32] Chen YY Zhai JB Shi T. Clinical effect meta analysis of acupuncture at renying point for primary hypertension. New Tradit Chin Med. 2017;49:184–8.

[33] Ma CY. Meta of acupuncture and moxibustion therapy for hypertension based on randomized controlled trials. Shandong Tradit Chin Med Univ. 2016.

[34] Ma ZQ Ge M. A meta-analysis of the safety and efficacy of acupuncture in treating hyperlipidemia. Henan province Traditl Chin Med. 2012;32:1398–401.

[35] Qian YX. Systematic evaluation of efficacy and safety of acupuncture in the treatment of essential hypertension. Northern Pharm. 2013;10:72–4.

[36] Shi LW Li Q Li XW. Systemic evaluation of acupuncture intervention in prediabetes. Shandong J Tradit Chin Med. 2018;37:282–8.

[37] Tang HZ. Systematic evaluation of acupuncture and moxibustion for essential hypertension. Chengdu Univ TCM. 2011.

[38] Wang F Pei J. Meta analysis of the effect of acupuncture on blood pressure variability regulation. Chin Insti Acupuncture Moxibustion. 2019:1046–52.

[39] Xia YY. A systematic evaluation of acupuncture obesity. Chengdu Univ TCM. 2015.

[40] Xiao GC. A meta analysis of the effect of acupuncture and moxibustion on essential hypertension. Guiyang Coll Tradit Chin Med. 2015.

[41] Xing CG Sun Z Ma YC. Meta analysis of the effect of acupuncture on islet function in type 2 diabetic patientset al. J Nanjing Univ Chin Med. 2015;31:397–400.

[42] Yang LF Huang LJ Li Y. Clinical effect of abdominal acupuncture on simple obesity meta analysis. Clin Study Tradit Chin Med. 2015;7:1–4.

[43] Yu H Tan J Han QJ. Effect of acupuncture on essential hypertension. Clin J Acupunct Moxibustion. 2013;29:39–45.

[44] Yu Z Ju CH Xu B. Evaluation of clinical randomized controlled trial of acupuncture for simple obesity. Shi Zhen Ntl Med. 2010;21:434–6.

[45] Zhang JP. Effect of acupuncture on fcmri brain function connection of taixi point in patients with essential hypertension. Southern Med Univ. 2017.

[46] Zhang L Zeng XT Tian GX. A meta analysis of the effect of acupuncture and antihypertensive drugs on essential hypertension. Chin J Evid Based Cardiovasc Med. 2017;9:1420–6.

[47] Zhang L Zeng XT Tian GX. Effect of acupuncture on essential hypertension and analysis of acupoint frequency. Liaoning J Tradit Chin Med. 2013;40:2115–9.

[48] Zhu T Ding L. Meta analysis of acupuncture at fengchi and quchi acupoints in the treatment of essential hypertension. Clin J Tradit Chin Med. 2018;30:461–5.

[49] Zhang YJ Shu ZJ Gao Y. Meta analysis of the effect of acupuncture and acupuncture on mild and moderate essential hypertension. Liaoning J Tradit Chin Med. 2014;41:1802–6.

[50] Zhao R Fu LX Xiong J. Systematic evaluation of the long-term effect of acupuncture on essential hypertension. Clin J Acupunct Moxibustion. 2011;27:46–51.

[51] Shan ZL Song AQ Qian SY. Meta analysis of clinical effect of electroacupuncture on simple obesity. Diet care. 2019;6:95–6.

[52] ogata J Yamanishi H Ishibashi-ueda H. Review: role of cerebral vessels in ischaemic injury of the brain. Neuropathol Appl Neurobiol. 2011;37:40–55. 21039751 10.1111/j.1365-2990.2010.01141.x

[53] Puja G Nirmal S Arunachalam M. Pharmacologic evidence for role of endothelial nitric oxide synthase in neuroprotective mechanism of ischemic postconditioning in mice. J Surg Res. 2014;188:349–60. 24439135 10.1016/j.jss.2013.12.015

[54] Peng JP. Study on the clinical efficacy and safety of cerebral angiography in acute ischemic cerebrovascular disease. World’s Latest Med Info Digest. 2017;17:12 + 14.

[55] Wang L. Clinical study of arterial thrombolysis combined with interventional therapy for senile patients with acute cerebrovascular occlusion.Modern diagnosis and treatment. 2017;28:1872–3.

[56] Hu J Pang WS Han J. Gualou Guizhi decoction reverses brain damage with cerebral ischemic stroke, multi-component directed multi-target to screen calcium-overload inhibitors using combination of molecular docking and protein–protein docking.. J Enzyme Inhib Med Chem. 2018;33:115–25. 29185359 10.1080/14756366.2017.1396457 PMC6009878

[57] Kim KA Shin D Kim JH. Role of autophagy in endothelial damage and blood-brain barrier disruption in ischemic stroke. Stroke. 2018;49:1571–9. 29724893 10.1161/STROKEAHA.117.017287

[58] Wilhelm I Nyúl-tóth A Kozma M. Role of pattern recognition receptors of the neurovascular unit in inflamm-aging. AJP Heart Circ Physiol. 2017;313:H1000–12. 10.1152/ajpheart.00106.2017 28801521

